# Diagnostic Performance of Serum Mac-2-Binding Protein Glycosylation Isomer as a Fibrosis Biomarker in Non-Obese and Obese Patients with MASLD

**DOI:** 10.3390/biomedicines13020415

**Published:** 2025-02-09

**Authors:** Prooksa Ananchuensook, Kamonchanok Moonlisarn, Bootsakorn Boonkaew, Chalermarat Bunchorntavakul, Pisit Tangkijvanich

**Affiliations:** 1 Division of Gastroenterology, Department of Medicine, Faculty of Medicine, Chulalongkorn University, Bangkok 10330, Thailand; prooksa.anan@gmail.com; 2 Academic Affair, Faculty of Medicine, Chulalongkorn University, Bangkok 10330, Thailand; 3 Center of Excellence in Hepatitis and Liver Cancer, Department of Biochemistry, Chulalongkorn University, Bangkok 10330, Thailand; 6570002830@student.chula.ac.th (K.M.); bootsakorn.b@gmail.com (B.B.); 4 Division of Gastroenterology, Department of Medicine, Rajavithi Hospital, Bangkok 10400, Thailand; dr.chalermrat@gmail.com

**Keywords:** liver fibrosis, metabolic dysfunction-associated steatotic liver disease, M2BPGi, magnetic resonance elastography, *PNPLA3*

## Abstract

**Background:** Serum mac-2-binding protein glycosylation isomer (M2BPGi) is a new biomarker for liver fibrosis. However, its performance in metabolic dysfunction-associated steatotic liver disease (MASLD), particularly in obese patients, remains to be explored. **Methods:** This study evaluated the role of M2BPGi in predicting liver fibrosis in 205 patients with MASLD using magnetic resonance elastography (MRE) as a reference. The performance of M2BPGi was compared to vibration-controlled transient elastography (VCTE), FIB-4, APRI, and NFS. The *PNPLA3*, *TM6SF2*, and *HSD17B13* polymorphisms were assessed by allelic discrimination assays. **Results:** The area under the ROC curves for VCTE, M2BPGi FIB-4, APRI, and NFS in differentiating significant fibrosis were 0.95 (95% CI; 0.91–0.98), 0.85 (0.79–0.92), 0.81 (0.74–0.89), 0.79 (0.71–0.87), and 0.80 (0.72–0.87) (all *p* < 0.001), respectively. The optimal cut-off values of M2BPGi in predicting significant fibrosis, advanced fibrosis, and cirrhosis were 0.82, 0.95, and 1.23 cut-off index (COI); yielding satisfactory sensitivity, specificity, and diagnostic accuracy. The performance of M2BPGi was consistent among subgroups according to BMI, while the AUROCs of FIB-4, APRI, and NFS were remarkably decreased in patients with BMI ≥ 30 kg/m^2^. Patients with the *PNPLA3* GG genotype had significantly higher M2BPGi than those with the CC/CG genotypes. In multivariate analysis, the independent factors associated with significant liver fibrosis were VCTE, M2BPGi, and *PNPLA3* rs738409. **Conclusions:** Our data demonstrated that serum M2BPGi accurately assessed liver fibrosis across different BMI, indicating that this biomarker could apply to non-obese and obese patients with MASLD in clinical settings.

## 1. Introduction

Metabolic dysfunction-associated steatotic liver disease (MASLD), formerly known as non-alcoholic fatty liver disease (NAFLD), is the most common chronic liver disease (CLD) worldwide, affecting approximately 30% of the global population [1]. The continuing liver injury of this disorder can lead to more severe forms, including steatohepatitis with or without liver fibrosis, cirrhosis, and—ultimately—hepatocellular carcinoma (HCC) [1]. Increasing evidence also indicates that progressive MASLD is an independent risk for developing cardiovascular disease, particularly in individuals with obesity and advanced liver disease [2]. Thus, accurate assessment of disease severity, particularly the extent of liver fibrosis, is essential for the management of patients with MASLD. At present, liver biopsy is the gold standard for diagnosis; however, this method is invasive, prone to sampling errors, and has poor acceptability, which limits its use in routine clinical settings [3]. Accordingly, several imaging techniques have been applied to measure the severity of liver fibrosis, including vibration-controlled transient elastography (VCTE) and magnetic resonance elastography (MRE). Currently, MRE and magnetic resonance imaging-proton density fat fraction (MRI-PDFF) are considered the most reliable non-invasive procedure for evaluating liver fibrosis and steatosis in patients with MASLD, respectively [4,5].

Serum-based biomarkers have also been developed in the past 20 years as an alternative to liver biopsy for evaluating the severity of liver fibrosis in CLD. These non-invasive biomarkers are mainly divided into simple biochemical tests or ‘indirect’ biomarkers—reflecting altered liver functions or liver inflammation—and ‘direct’ biomarkers, which represent the circulating components of extracellular matrix re-modeling during hepatic fibrogenesis [6]. The widely used fibrosis models in patients with MASLD that include a combination of indirect biomarkers are the fibrosis-4 (FIB-4) index, aspartate aminotransferase (AST)/platelet ratio index (APRI), and NAFLD fibrosis score (NFS) [6]. Although these fibrosis models are easy to use in routine practice, the tests are not liver-specific, and their performance may be affected by some clinical parameters, particularly high body mass index (BMI) [7]. Notably, it has been shown that the accuracy of FIB-4 and NFS for diagnosing advanced fibrosis in obese individuals was significantly lower than that of patients with normal weight [7].

Among ‘direct biomarkers’, serum mac-2-binding protein glycosylation isomer (M2BPGi) measured by glycan-based immunoassays has emerged as an accurate liver-specific biomarker for assessing liver fibrosis stages resulting from various CLDs, particularly chronic hepatitis C and B, as well as autoimmune-related inflammatory disorders such as autoimmune hepatitis (AIH) and primary biliary cholangitis (PBC) [8]. This novel biomarker has also helped monitor disease progression and predict HCC development in patients with chronic viral hepatitis [8]. Additionally, the clinical utility of M2BPGi as a fibrosis marker has been explored in patients with MASLD, and the results suggest that this novel biomarker is reliable for distinguishing liver fibrosis stages [9], as well as cirrhosis [10,11]. Moreover, a recent study showed that higher serum M2BPGi levels were significantly related to an increased risk of diabetes in Japanese individuals [12]. Despite these findings, the available data are still limited and have inconsistencies among reports that need further investigation, particularly its diagnostic accuracy regarding different BMIs of patients.

This cross-sectional study aimed to examine the clinical utility of serum M2BPGi in a well-characterized cohort of Thai patients with MASLD. Using MRE as the reference for assessing fibrosis staging, the diagnostic performance of serum M2BPGi was directly compared with VCTE and conventional fibrosis models, including FIB-4, APRI, and NFS. In this study, we also investigated various clinical characteristics that might impact the severity of fibrosis, including obesity, type 2 diabetes (T2DM), and single nucleotide polymorphisms (SNPs), which are important factors associated with the development and progression of MASLD [13].

## 2. Materials and Methods

### 2.1. Patients

Between 2022 and 2024, 205 Thai patients diagnosed with MASLD were enrolled in this cross-sectional cohort at the King Chulalongkorn Memorial Hospital, Thailand. The Institute Ethics Committee approved the study (IRB No. 981-64), and the study was conducted following the Declaration of Helsinki and the principles of good clinical practice. Written informed consent was obtained from patients, and their medical records were reviewed before enrollment. For all participants, anthropometric variables—including weight, height, and BMI—were measured. According to the Asian-BMI categorization, individuals with BMI of ≤24.9, 25–29.9, and ≥30 kg/m^2^ were classified as non-obese, obese class I, and obese class II, respectively [14].

The inclusion criteria were patients aged ≥18 years diagnosed with liver steatosis based on MRI-PDFF grade ≥1 (defined as MRI-PDFF ≥5.4%) [5]. Exclusion criteria were: (1) concomitant other chronic liver diseases such as chronic viral hepatitis, Wilson’s disease, autoimmune hepatitis, and primary biliary cirrhosis; (2) presence of cirrhotic complications (e.g., ascites) or evidence of HCC; (3) presence of other conditions causing secondary steatosis such as human immunodeficiency virus (HIV) infection; (4) known active malignancies or severe health conditions; (5) current significant alcohol misuse or history of alcohol consumption (≥30 g for men and ≥20 g for women).

### 2.2. Laboratory Analyses

Serum biochemical parameters, including AST, alanine aminotransferase (ALT), alkaline phosphatase (ALP), albumin, total cholesterol, triglyceride, high-density lipoprotein (HDL) cholesterol, low-density lipoprotein (LDL) cholesterol, fasting plasma glucose (FPG), hemoglobin A1C (HbA1C) test, and platelet count were measured using a conventional automated analyzer. The FIB-4 index was calculated using the following formula: age (years) × AST [U/L]/platelets [10^9^/L] × ALT [U/L])^1/2^. The APRI score was calculated by the formula: AST [U/L] × 100/platelets [10^9^/L] × ALT [upper limit of normal] [U/L]). The NFS was based on the following calculation: −1.675 + 0.037 × Age (years) + 0.094 × BMI (kg/m^2^) + 1.13 × impaired glucose tolerance/diabetes (yes = 1, no = 0) + 0.99 × (AST/ALT ratio) − 0.013 × platelets (×10^9^/L) − 0.66 × serum albumin (g/dL).

Serum samples for M2BPGi collected from the patients were stored at −80 °C until analysis. The biomarker was measured by lectin-antibody sandwich immunoassay using a fully automatic immune analyzer (HISCL-2000i, Sysmex, Hyogo, Japan). M2BPGi level was then calculated by the following equation: cut-off index (COI) = ([M2BPGi]sample − [M2BPGi]negative controls)/[M2BPGi]positive controls − [M2BPGi]negative control]), as previously described [15].

### 2.3. Imaging Studies for Liver Stiffness and Steatosis

Liver stiffness measurement (LSM) and controlled attenuation parameter (CAP) were measured using VCTE (Echosens, Paris, France) with M-probe and XL-probe as appropriate. The procedure was based on at least 10 validated measurements with a success rate of over 60% and an interquartile range of less than 30% [16] MRE and MRI-PDFF were conducted by the MRI system Philips Ingenia at 3.0 T (Philips Healthcare, Best, The Netherlands). The cut-off values for fibrosis ≥F1, ≥F2, ≥F3, and F4 by MRE measurement were 2.6, 3.0, 3.6, and 4.7 kPa, respectively, based on a systematic review and meta-analysis in MASLD [4]. For the measurement of MRI-PDFF, the cut-off values for diagnosing steatosis grades ≥1, ≥2, and ≥3 were 5.4%, 15.4%, and 20.4%, respectively [5].

### 2.4. DNA Extraction and SNP Genotyping

DNA was extracted from peripheral blood mononuclear cells (PBMCs) using the phenol-chloroform isoamyl alcohol technique, and its quantity and quality measurement was performed by a DeNovix™ UV-Vis spectrophotometer. The DNA samples were then stored at −80 °C until analysis. For genotyping of the *patatin-like phospholipase domain containing 3 (PNPLA3) rs738409*, *transmembrane 6 superfamily member 2 (TM6SF2) rs58542926*, and *17β-hydroxysteroid dehydrogenase 13 (HSD17B13) rs6834314* genes, allelic discrimination with TaqMan Probe Real-Time PCR Assays (ThermoFisher Scientific, Waltham, MA, USA), and fluorescent signals (FAM and VIC) detection were applied as previously described [17]. To confirm the accuracy of interpretation, positive and negative controls were involved in each experiment, and the allelic discrimination plot was assessed by the QuantStudio™ 3 Real-Time PCR System (ThermoFisher Scientific, USA).

### 2.5. Statistical Analyses

Statistical analyses were performed using the IBM SPSS software version 23.0 (IBM, Chicago, IL, USA). Data were presented as percentages or mean ± standard deviation (SD). Comparisons between groups were assessed by analysis of variance and the Student’s *t*-test or nonparametric Mann–Whitney U test when appropriate. Based on MRE as the reference, the diagnostic performances of VCTE, M2BPGi, FIB-4, APRI, and NFS were calculated using the receiver operator characteristics (ROC) curves. The area under the ROC (AUROC) was compared, and the cut-off values were determined, to predict the fibrosis stage. The diagnostic performance was also determined regarding sensitivity, specificity, positive predictive value (PPV), and negative predictive value (NPV). Spearman’s rank test was applied to evaluate the correlation of serum M2BPGi with other parameters. Linear regression analysis was applied to test variables associated with M2BPGi. Univariate and multivariable analyses were evaluated using binary logistic regression to determine parameters related to F2–F4 fibrosis. A *p*-value of <0.05 was considered as statistically significant.

## 3. Results

### 3.1. Patient Characteristics

A total of 205 patients with MASLD were recruited in this study. Their clinical and laboratory characteristics are shown in Table 1. The mean age of the patients was 57.0 ± 13.4 years, and 105 (51.2%) were men. There were 65 (31.7%), 89 (43.4%), and 72 (35.1%) patients with a history of type 2 diabetes (T2DM), hypertension, and dyslipidemia, respectively. There were 150 (73.2%) patients classified as obese, and the average BMI of all patients was 27.8 ± 4.6 kg/m^2^. The mean values of MRE and MRI-PDFF were 2.9 ± 1.2 kPa and 12.2 ± 7.5%. Sixty-two (30.2%) patients had significant fibrosis (F2) or more, defined as MRE ≥3.0 kPa.

### 3.2. Diagnostic Performance of VCTE and Serum Biomarkers

**Figure 1:**
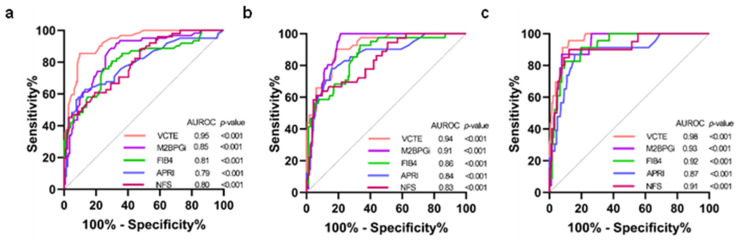
Comparison of AUROCs between VCTE and serum biomarkers (**a**) F0–F1 vs. F2–F4; (**b**) F0–F2 vs. F3–F4; (**c**) F0–F3 vs. F4. AUROCs, area under the receiver operator curves; VCTE, vibration-controlled transient elastography; M2BPGi, serum mac-2-binding protein glycosylation isomer; FIB-4, fibrosis-4 index; APRI, aspartate aminotransferase/platelet ratio index; NFS, non-alcoholic fatty liver disease fibrosis score; COI, cut-off index.

The diagnostic performance of VCTE, M2BPGi, FIB-4, APRI, and NFS was calculated by the AUROCs using MRE as the reference method for defining fibrosis stages (Figure 1). The AUROCs for VCTE in distinguishing significant fibrosis (≥F2), advanced fibrosis (≥F3), and cirrhosis (F4) were 0.95 [95% confident interval (CI); 0.91–0.98, *p* < 0.001], 0.94 (0.90–0.98, *p* < 0.001) and 0.98 (0.95–1.00, *p* < 0.001), respectively. The corresponding figures for M2BPGi were 0.85 (0.79–0.92, *p* < 0.001), 0.91 (0.87–0.96, *p* < 0.001), and 0.93 (0.88–10.98, *p* < 0.001), respectively. For FIB-4, the corresponding values were 0.81 (0.74–0.89, *p* < 0.001), 0.86 (0.79–0.93, *p* < 0.001), and 0.92 (0.88–0.97, *p* < 0.001), respectively. The corresponding figures for APRI were 0.79 (0.71–0.87, *p* < 0.001), 0.84 (0.76–0.92, *p* < 0.001), and 0.87 (0.78–10.96, *p* < 0.001), respectively. Regarding NFS, the corresponding values were 0.80 (0.72–0.87, *p* < 0.001), 0.83 (0.76–0.91, *p* < 0.001), and 0.91 (0.84–0.98, *p* < 0.001), respectively.

### 3.3. The Performance of M2BPGi in Assessing Fibrosis Stages

**Figure 2:**
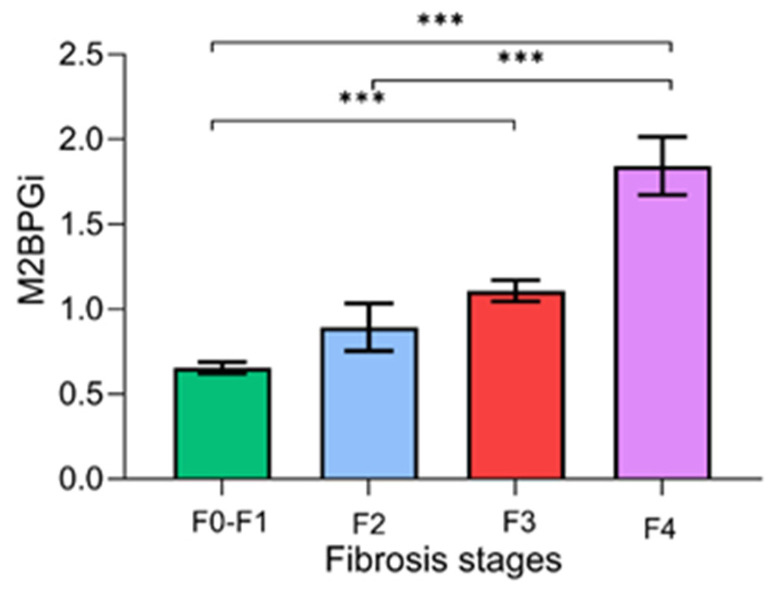
Serum M2BPGi values for each fibrosis stage. Data are presented as mean ± S.E.M. *** *p*-value < 0.001.

Figure 2 demonstrates the serum M2BPGi values for each fibrosis stage. There were significant differences in the mean levels of M2BPGi between the F0–F1 and F2–F4 stages (0.66 ± 0.40 vs. 1.31 ± 0.76 COI, *p* < 0.001), between F0–F2 and F3–F4 (0.69 ± 0.44 vs. 1.52 ± 0.73 COI, *p* < 0.001), and between F0–F3 and F4 (0.73 ± 0.45 vs. 1.84 ± 0.82 COI, *p* < 0.001).

Table 2 demonstrates the optimal cut-off value, sensitivity, specificity, PPV, and NPV of M2BPGi for each fibrosis stage. The AUROCs were 0.85, 0.91, and 0.93 for ≥F2, ≥F3, and F4, respectively. The optimal cut-off values that best predicted the corresponding fibrosis stages were 0.82, 0.95, and 1.23, respectively. Overall, M2BPGi was considered a reliable single biomarker for diagnosing significant fibrosis, advanced fibrosis, and cirrhosis.

### 3.4. Relationship Between M2BPGi Levels and Clinical Parameters

The relationship between serum M2BPGi levels and clinical parameters was also assessed. There was a positive correlation found between M2BPGi and age (r = 0.305, *p* < 0.001) and AST (r = 0.300, *p* < 0.001). In contrast, a negative correlation was found between M2BPGi and platelet counts (r = −0.274, *p* < 0.001) and albumin (r = −0.348, *p* < 0.001). There was no significant correlation between M2BPGi and other clinical parameters (sex, BMI, FPG, lipid profiles, creatinine, bilirubin, ALT, and ALP).

Among fibrosis markers, M2BPGi level was positively correlated with MRE (r = 0.558, *p* < 0.001), VCTE (r = 0.391, *p* < 0.001), FIB-4 (r = 0.439, *p* < 0.001), APRI (r = 0.383, *p* < 0.001), and NFS (r = 0.483, *p* < 0.001). However, there was no correlation between M2BPGi level and MRI-PDFF (r = −0.116, *p* = 0.102).

### 3.5. Performance of VCTE and Serum Biomarkers According to Age and BMI

We further determined the AUROCs for diagnosing significant fibrosis for VCTE and serum biomarkers compared with the reference method based on MRE in subgroups of patients, according to age and BMI (Table 3). With VCTE, the lowest AUROC was found for obese patients with BMI ≥30 kg/m^2^. Similar patterns among these patients with obese class II were observed for FIB-4, APRI, and NFS. However, the AUROCs for M2BPGi were relatively consistent among subgroups regardless of the patient’s age and BMI.

### 3.6. Distributions of SNPs According to Fibrosis Stages

In this cohort, the genotype frequencies of SNPs, including *PNPLA3 rs738409*, *TM6SF2 rs58542926*, and *HSD17B13 rs6834314*, were also investigated. The frequencies of *PNPLA3* (CC/CG/GG) were 53 (25.9%)/74 (36.1%)/78 (38.0%); while the distributions of *TM6SF2* (CC/CT/TT) were 156 (76.1%)/40 (19.5%)/9 (4.4%); and those of *HSD17B13* (AA/AG/GG) were 82 (40.0%)/87 (42.4%)/22 (10.7%), and 14 (6.8%) samples were unclassified. Patients with F2–F4 fibrosis had a higher frequency of *PNPLA3* GG genotype than patients with F0–F1 (56.5% vs. 30.1%, *p* = 0.001]. However, the frequencies of *TM6SF2* CT+TT in the F2–F4 vs. F0–F1 groups were not significant (27.4% vs. 22.4%, *p* = 0.477], which was similar to the distributions of *HSD17B13* AG+GG between the corresponding groups (64.2% vs. 54.3%, *p* = 0.255].

Interestingly, patients harboring *the PNPLA3 GG* genotype had a significantly higher M2BPGi than those with *PNPLA3* CC+CG genotypes (0.99 ± 0.81 vs. 0.77 ± 0.43, *p* = 0.029). Similar trends regarding the *PNPLA3* GG vs. CC+CG genotypes were also observed among the imaging modalities and other serum fibrosis biomarkers; MRE (3.1 ± 1.3 vs. 2.7 ± 1.1 kPa, *p* = 0.023), VCTE (11.2 ± 11.5 vs. 9.3 ± 7.1 kPa, *p* = 0.188), FIB-4 (1.73 ± 1.64 vs. 1.30 ± 1.06, *p* = 0.044), APRI (0.40 ± 0.37 vs. 0.35 ± 0.32, *p* = 0.334), and NFS (−1.17 ± 1.88 vs. −1.76 ± 1.64, *p* = 0.045).

### 3.7. Factors Predicted Significant Fibrosis

We further investigated whether the parameters in our cohort were independently associated with significant fibrosis (≥F2). The factors included age, gender, BMI, T2DM, HT, DLP, AST, ALT, platelet count, liver steatosis grade, *PNPLA3 rs738409*, *TM6SF2 rs58542926*, and *HSD17B13 rs6834314;* as well as VCTE, M2BPGi, FIB-4, APRI, and NFS. In univariate analysis, parameters associated with significant fibrosis were age, the presence of T2DM and HT, AST, platelet, steatosis grade, *PNPLA3 rs738409*, VCTE, and all serum fibrosis biomarkers. In multivariate analysis, only *PNPLA3 rs738409*, VCTE, and serum M2BPGi were independently associated with significant fibrosis (Table 4).

When combining analysis of *PNPLA3* and M2BPGi, our data showed that 0/81 (0%) patients harboring the *PNPLA3* CC+CG genotypes with low M2BPGi (<0.82 COI) had significant fibrosis (≥F2), while 22/81 (27.2%) patients with either *PNPLA3* GG or high M2BPGi level (≥0.82 COI) had significant fibrosis. Additionally, 28/43 (65.1%) patients who exhibited high M2BPGi levels and also harbored the *PNPLA3* GG had significant fibrosis (*p* < 0.001).

## 4. Discussion

MASLD, characterized by excess intrahepatic fat accumulation accompanied by metabolic dysregulation, is now considered a multisystem disease and a leading global health concern. As the degree of liver fibrosis is a major determining factor for disease outcomes and overall survival, it is essential to identify appropriate non-invasive biomarkers for accurate assessment in patients with MASLD, particularly differentiating F2–F4 from mild fibrosis stages [18]. In this study, our data demonstrated that serum M2BPGi performed well in distinguishing liver fibrosis stages F0–F1 vs. F2–F4. Although the AUROC of serum M2BPGi in detecting fibrosis ≥F2 was inferior to that of VCTE, its diagnostic performance was superior to those of FIB-4, APRI, and NFS. Additionally, multivariate regression analysis showed that serum M2BPGi levels were independently associated with the fibrosis stage (F2–F4). In particular, the performance of M2BPGi was not influenced by the differing BMI as patients within the non-obese, obese class I, and obese class II groups exhibited similar AUROCs. Our data suggest that M2BPGi could be a suitable serum-based biomarker to differentiate early from significant fibrosis in patients with MASLD, regardless of different BMI. Although previous reports indicated that BMI was not an independent factor influencing M2BPGi levels [10,19], these data did not directly address the diagnostic accuracy of this biomarker compared to other simple serum algorithms across different BMIs, as demonstrated in our study.

Current evidence has indicated that MRE is the best alternative to liver biopsy due to its excellent accuracy in diagnosing and stratifying liver fibrosis; thus, this imaging modality was selected as the reference in our study. Based on a pooled data analysis from individual participants with biopsy-proven MASLD, MRE displays a significantly higher accuracy than VCTE in detecting each fibrosis stage [4]. For example, MRE has AUROCs of 0.92, 0.93, and 0.94 for the prediction of ≥F2, ≥F3, and F4 fibrosis stages, respectively; while the corresponding figures for VCTE are 0.87, 0.84, and 0.84, respectively. Furthermore, using MRE allows the visualization and assessment of the entire liver, rather than sampling small hepatic regions as applied by VCTE. Operator skills might also affect the diagnostic success rate of VCTE, indicating its limitations, particularly in examining individuals with obesity. Additionally, a meta-analysis indicates that BMI minimally influences MRE cut-off values for assessing liver fibrosis stage [20]. On the contrary, it has been shown that VCTE with either M and XL probes displays lower diagnostic accuracy for F2–F4 and F3–F4 fibrosis in patients with BMI ≥30 kg/m^2^ [21]. In agreement with these observations, our results also demonstrated that the AUROCs for detecting F2–F4 fibrosis for VCTE were comparable between patients with BMI ≤24.9 and 25–29.9 kg/m^2^. Still, they noticeably declined in patients with BMI ≥30 kg/m^2^.

M2BPGi is a highly glycosylated form of the mac-2-binding protein (M2BP) secreted by hepatic stellate cells (HSCs), and its expression is closely related to HSC activation [22]. Thus, measuring this extracellular matrix protein in the serum could reflect the fibrogenic process irrespective of the etiologic factors of CLD [22,23]. Current evidence has shown that serum M2BPGi is a promising biomarker that correlates well with the severity of liver fibrosis in various CLDs, such as chronic viral hepatitis, primary biliary cholangitis, and autoimmune hepatitis [8]. Regarding MASLD, previous studies also showed that serum M2BPGi increased with the progression of fibrosis stage, particularly in significant fibrosis to cirrhosis [9,10,11,19,24]. For instance, a Japanese study of patients with biopsy-proven MASLD demonstrated a stepwise increase of serum M2BPGi values with progressive fibrosis, which displayed a superior AUROC for the diagnosis compared with the FIB-4, APRI, and NFS, among other biomarkers [10]. In that study, the AUROCs for detecting ≥F2 and ≥F3 fibrosis were 0.84 and 0.88, respectively. These data aligned with our results, indicating that serum M2BPGi could predict F2–F4 fibrosis in patients with MASLD, as the AUROCs in our study for ≥F2, ≥F3 and F4 fibrosis were 0.85, 0.91, and 0.93, respectively. Moreover, a meta-analysis indicated that the overall AUROCs of serum M2BPGi for detecting significant fibrosis, advanced fibrosis, and cirrhosis from any etiologic factor of CLD were 0.79, 0.82 and 0.88, respectively [25].

A recent report also demonstrated that serum M2BPGi was precise in estimating severe liver stiffness assessed by MRE in patients with MASLD with a single cut-off value unrelated to patients’ age [26]. In this context, our study showed comparable AUROCs, demonstrating that age was not a confounder for M2BPGi measurement. We further explored the impact of BMI in association with the performance of each serum biomarker in predicting significant fibrosis. Our data displayed that the AUROCs for serum M2BPGi in differentiating F0–F1 vs. F2–F4 did not change with the BMI altered. In contrast, the AUROCs for the FIB-4, APRI, and NFS remarkably decreased in patients with BMI ≥30 kg/m^2^, which agreed with previous data indicating that these serum fibrosis models appear to be less accurate in patients with obesity [7]. These findings are considered necessary given that obesity is highly prevalent in patients with MASLD and could be a significant limitation of their use in clinical practice. In our study, for example, approximately 75% and 25% of patients were classified as obese in class I and II, respectively. Indeed, the proportion of patients with BMI ≥25 kg/m^2^ (75%) was slightly higher than that of the Asian prevalence in a recent meta-analysis (66%) [27], probably due to the older mean age of patients in our cohort (57.0 vs. 52.1 years).

Of note, serum M2BPGi levels might vary significantly depending on the underlying etiologies of CLD. For example, the cut-off levels of M2BPGi in patients with chronic viral hepatitis C are mostly higher than those of patients with MASLD in the same fibrosis stages [28]. In the meta-analysis of serum M2BPGi—which covered broad etiologies of CLD—the optimal cut-off levels for ≥F2, ≥F3, and F4 fibrosis stages in MASLD were 0.90–1.00, 0.94–1.10, and 1.46–1.60 COI, respectively [28]. The corresponding cut-off values in our study were 0.82, 0.95, and 1.23 COI, respectively. In fact, the cut-off level for ≥F2 fibrosis in our study was very similar to a previous Japanese report (0.83 COI) based on histopathologically proven MASLD [29]. Thus, it appears that the cut-off threshold for each fibrosis stage might differ from study to study, which could probably be related to several factors, such as the populations in studied cohorts and the methods for defining the severity of liver fibrosis.

Our study also highlighted the role of the *PNPLA3* rs738409 genotype in association with significant fibrosis. In contrast, *TM6SF2 rs58542926* and *HSD17B13* rs6834314 did not relate to progressive fibrosis. The *PNPLA3* polymorphism has been documented from genome-wide association studies as increased steatosis susceptibility and is currently the most robust genetic determinant in MASLD [30]. Indeed, *PNPLA3* GG and CG accounted for most patients in our cohort (38.0% and 36.1%, respectively), reflecting the enrichment of the risk genotypes in individuals with progressive liver disease. These results agreed with previous data indicating an increased risk of this polymorphism on fibrosis progression in patients with MASLD [31]. Interestingly, our data also demonstrated that serum levels of M2BPGi in patients with the *PNPLA3 GG* genotype were significantly higher than those with the CC/CG genotypes. The mechanisms by which this polymorphism links to increased M2BPGi levels are unclear. However, it has been shown in vitro that the function of the PNPLA3 protein is necessary for HSC activation, and this variant could, in turn, promote the profibrogenic characteristics of HSCs, resulting in an increased risk of fibrosis progression [32]. As HSCs are the primary source of M2BP production, it is speculated that high M2BP expression might be linked to enhanced HSC activation in patients carrying the GG genotype.

This study has some limitations. First, we evaluated the usefulness of the serum M2BPGi in a cross-sectional study. However, the role of this biomarker in monitoring the natural history and predicting the clinical outcomes of MASLD remains unknown. In this context, a previous study indicated that the biomarker could predict HCC development in patients with MASLD unrelated to its levels as a fibrosis biomarker [33]. Another limitation was that this serum M2BPGi positively correlated, albeit weakly, with serum AST level. This finding was in line with a recent report demonstrating that quantitative measurement of serum M2BPGi might depend on liver inflammation and fibrosis, regardless of the etiologic causes of CLD [34]. Thus, falsely increased M2BPGi levels might be affected by the activity of liver inflammation, emphasizing the need for interpretation. Additionally, increasing or decreasing its circulating levels might involve other related molecular pathways—including M2BP mRNA expression, post-translational modifications, and glycosylation—that could compromise diagnostic accuracy [23]. Moreover, our study included only Thai patients, which might not be generalizable to other ethnic populations and diverse clinical circumstances. Finally, the sample size of individuals with F2–F4 was relatively small, which could reflect the lesser distribution of significant fibrosis to cirrhosis typically found in real-life circumstances. For example, recent real-world data from a large European report revealed that the prevalence of significant fibrosis defined by FIB-4 was approximately 30–35%, similar to our cohort [35]. Accordingly, the subgroup analysis of M2BPGi performance, such as stratified by BMI and the PNPLA3 genotypes, might lead to inadequate statistical power as a type II error due to our small sample size.

## 5. Conclusions

In conclusion, as the global incidence of MASLD increases, finding non-invasive biomarkers for accurately predicting the severity of liver fibrosis is becoming critically important. Although fibrosis stages based on simple serum algorithms are practical and not expensive, their results could be negatively affected by obesity. Our data demonstrated that measuring serum M2BPGi levels could accurately assess liver fibrosis in patients with MASLD, particularly in patients with F2–F4 fibrosis, independently of BMI. Since obesity and metabolic disturbance are closely related to MASLD development, serum M2BPGi could be used as a promising fibrosis biomarker for MASLD in clinical settings. However, additional prospective studies with a larger sample size on the diagnostic role of serum M2BPGi, especially among obese patients with MASLD, are warranted.

## Figures and Tables

**Table 1:** Clinical characteristics of patients in this study.

Characteristics	MASLD (n = 205)
Age (years)	57.0 ± 13.4
Gender (male/female)	51.2 (51.2)/48.8 (48.8)
Body mass index (kg/m^2^) (<25.0/25.0–29.9/≥30.0)	55 (26.8)/98 (47.8)/52 (25.4)
Presence of type 2 diabetes	65 (31.7)
Presence of hypertension	89 (43.4)
Presence of dyslipidemia	72 (35.1)
Creatinine (mg/dL)	1.0 ± 1.2
Hemoglobin (g/dL)	13.8 ± 1.6
White blood count (10^3^/µL)	7.0 ± 2.0
Platelet count (10^3^/µL)	248.2 ± 72.1
Total bilirubin (mg/dL)	0.7 ± 0.3
Serum albumin (g/dL)	4.4 ± 0.3
Aspartate aminotransferase (IU/L)	28.5 ± 13.8
Alanine aminotransferase (IU/L)	36.4 ± 22.4
Alkaline phosphatase (IU/L)	73.6 ± 27.1
Magnetic resonance elastography (kPa)	2.9 ± 1.2
Proton density fat fraction (%)	12.2 ± 7.5
Liver fibrosis stage (F0–1/F2/F3/F4)	143 (69.8)/21 (10.2)/18 (8.8)/23 (11.2)
Liver steatosis grade (S1/S2/S3)	140 (68.3)/30 (14.6)/35 (17.1)

Data are presented as n (%)/mean ± SD.

**Table 2:** M2BPGi values for the assessment of fibrosis stages.

Fibrosis Stage	AUROCs	COI	Sensitivity (%)	Specificity (%)	PPV (%)	NPV (%)	Accuracy (%)
≥F2	0.85	0.82	74.2	79.0	60.5	87.6	77.6
≥F3	0.91	0.95	80.5	86.6	60.0	94.7	85.4
F4	0.93	1.23	87.0	92.3	58.8	98.3	91.7

Abbreviations: AUROCs, area under the receiver operator curves; COI, cut-off index; PPV, positive predictive value; NPV, negative predictive value.

**Table 3:** AUROCs of fibrosis biomarkers for significant fibrosis (≥F2) according to age and BMI.

	VCTE	M2BPGi	FIB-4	APRI	NFS
Age < 60	0.95 (0.89–1.00)	0.85 (0.76–0.94)	0.78 (0.65–0.92)	0.74 (0.58–0.90)	0.88 (0.80–0.96)
Age ≥ 60	0.94 (0.89–0.99)	0.84 (0.75–0.93)	0.80 (0.70–0.90)	0.80 (0.69–0.90)	0.70 (0.58–0.83)
BMI < 25	0.98 (0.95–1.00)	0.85 (0.74–0.97)	0.86 (0.75–0.97)	0.76 (0.60–0.93)	0.85 (0.73–0.97)
BMI = 25.0–29.9	0.95 (0.89–1.00)	0.86 (0.75–0.96)	0.84 (0.75–0.94)	0.83 (0.73–0.93)	0.82 (0.72–0.92)
BMI ≥ 30	0.89 (0.77–1.00)	0.88 (0.77–0.99)	0.74 (0.56–0.92)	0.74 (0.54–0.93)	0.70 (0.53–0.88)

AUROCs (95% confidence interval). Abbreviations: AUROCs, area under the receiver operator curves; BMI, body mass index; VCTE, vibration-controlled transient elastography; M2BPGi, serum mac-2-binding protein glycosylation isomer; FIB-4, fibrosis-4 index; APRI, aspartate aminotransferase/platelet ratio index; NFS, non-alcoholic fatty liver disease fibrosis score.

**Table 4:** Factors associated with significant fibrosis (≥F2).

Factors	Category	Univariate Analysis		Multivariate Analysis	
		OR (95% CI)	*p*-Value	OR (95% CI)	*p*-Value
Age (years)	≥60 vs. <60	2.19 (1.19–4.03)	0.012 *	1.07 (0.18–6.38)	0.943
Gender	Male vs. Female	1.29 (0.71–2.35)	0.402		
BMI (kg/m^2^)	≥25 vs. <25	1.08 (0.55–2.12)	0.828		
Diabetes	Yes vs. No	3.56 (1.89–6.69)	<0.001 *	1.39 (0.33–5.74)	0.653
Hypertension	Yes vs. No	3.15 (1.70–5.86)	<0.001 *	1.52 (0.36–6.36)	0.568
Dyslipidemia	Yes vs. No	0.83 (0.44–1.57)	0.572		
Aspartate aminotransferase (IU/L)	≥40 vs. <40	8.94 (3.51–22.75)	<0.001 *	1.66 (0.09–30.36)	0.733
Alanine aminotransferase (IU/L)	≥40 vs. <40	1.29 (0.68–2.43)	0.432		
Platelet count (10^9^/L)	<150 vs. ≥150	6.10 (1.97–18.84)	0.002 *	2.12 (0.13–34.64)	0.599
Liver steatosis grade	S2+S3 vs. S1	2.07 (1.03–4.18)	0.042 *	1.09 (0.24–4.97)	0.909
*PNPLA3* rs738409	GG vs. CC+CG	3.02 (1.63–5.58)	<0.001 *	5.00 (1.15–21.81)	0.032 *
*TM6SF2* rs58542926	CT+TT vs. CC	1.31 (0.66–2.59)	0.438		
*HSD17B13* rs6834314	AA vs. AG+GG	1.50 (0.78–2.89)	0.222		
VCTE (kPa)	≥7.6 vs. <7.6	28.39 (11.76–68.53)	<0.001 *	21.78 (4.97–95.47)	<0.001 *
M2BPGi (COI)	≥0.82 vs. <0.82	25.19 (10.49–60.47)	<0.001 *	11.16 (2.55–48.95)	0.001 *
FIB-4	≥1.30 vs. <1.30	8.36 (4.20–16.62)	<0.001 *	3.06 (0.49–19.22)	0.233
APRI	≥0.50 vs. <0.50	16.95 (6.83–42.06)	<0.001 *	0.87 (0.04–16.95)	0.925
NFS	≥1.455 vs. <1.455	4.20 (2.03–8.70)	<0.001 *	1.01 (0.23–4.52)	0.987

Data expressed as odds ratio (OR) and 95% confidence intervals (CI) * *p*-value < 0.05. Abbreviations: BMI, body mass index; VCTE, vibration-controlled transient elastography; M2BPGi, serum mac-2-binding protein glycosylation isomer; FIB-4, fibrosis-4 index; APRI, as-partate aminotransferase/platelet ratio index; NFS, non-alcoholic fatty liver disease fibrosis score; COI, cut-off index.

## Data Availability

The datasets used and/or analyzed during the current study are available from the corresponding author on reasonable request.

## Abbreviations

The following abbreviations are used in this manuscript:

MASLD	Metabolic dysfunction-associated steatotic liver disease
NAFLD	Non-alcoholic fatty liver disease
CLD	Chronic liver disease
HCC	Hepatocellular carcinoma
M2BPGi	Serum mac-2-binding protein glycosylation isomer
APRI	Aspartate aminotransferase/platelet ratio index
BMI	Body mass index
MRE	Magnetic resonance elastography
VCTE	Vibration-controlled transient elastography
MRI-PDFF	Magnetic resonance imaging-proton density fat fraction
NFS	Non-alcoholic fatty liver disease fibrosis score
AUROCs	Area under the receiver operator curves
COI	Cut-off index
PPV	Positive predictive value
NPV	Negative predictive value
OR	Odds ratio
CI	Confidence interval
